# Supplementary material for: Neuroimaging Changes in the Sensorimotor Network and Visual Network in Bipolar Disorder and Their Relationship with Genetic Characteristics
Source: Biomedicines. 2025 Apr 8;13(4):898. doi: 10.3390/biomedicines13040898 (PMC12025223; doi:10.3390/biomedicines13040898)
Supplement: Supplementary file 1 [file biomedicines-13-00898-s001.zip › biomedicines-3440040-supplementary.pdf]

Neuroimaging Changes in the Sensorimotor Network and Visual Network in Bipolar Disorder and Their Relationship with Genetic Characteristics

Chunguo Zhang[a#], Yiding Han[b#], Haohao Yan[b], Yangpan Ou[b], Jiaquan Liang[a], Wei Huang[a], Xiaoling Li[a], Chaohua Tang[a], Jinbing Xu[a], Guojun Xie[a*], Wenbin Guo[b*]

Affiliation/address:

a. Department of Psychiatry, The Third People's Hospital of Foshan, Foshan, Guangdong 528000, China.

b. Department of Psychiatry, National Clinical Research Center for Mental Disorders, and National Center for Mental Disorders, The Second Xiangya Hospital of Central South University, Changsha 410011, Hunan, China.

#: Chunguo Zhang and Yiding Han contributed equally to this work.

***Correspondence:**

Wenbin Guo

Department of Psychiatry, National Clinical Research Center for Mental Disorders, and National Center for Mental Disorders, The Second Xiangya Hospital of Central South University, Changsha 410011, Hunan, China.

Email: guowenbin76@csu.edu.cn

Guojun Xie

Department of Psychiatry, The Third People's Hospital of Foshan, Foshan, Guangdong 528000, China.

Email: xiegjfs@126.com

Table S1. The coordinates of region of interests.

Regions of Interest (ROIs)		MNI (x, y, z)
Sensorimotor Network	ROI1: Lateral Sensorimotor Network (L)	-55 -12 29
	ROI2: Lateral Sensorimotor Network (R)	56 -10 29
	ROI3: Superior Sensorimotor Network	0 -31 67
Visual Network	ROI4: Medial Visual Network	2 -79 12
	ROI5: Occipital Visual Network	0 -93 -4
	ROI6: Lateral Visual Network (L)	-37 -79 10

	ROI7: Lateral Visual Network (R)	38 -72 13

MNI = Montreal Neurologic Institute; R = right; L = left.

Table S2. Demography and clinical characteristics

Variables	Patients (Mean ± SD, n=77)	Controls (Mean ± SD, n=83)	*p*-value
Age (years)	30.44±8.88	34.84±11.93	0.074[a]
Gender (male/female)	28/49	33/50	0.659[b]
Years of education (years)	12.99±3.06	13.24±3.67	0.096[a]
Illness duration (months)	88.35±71.65		
Manic/Depressive/Hypomanic/Mixed episodes	59/18/0/0		
BMI (kg/m^2)	23.62±4.33	22.83±3.70	0.304[a]
TSH (mIU/L)	1.69±1.16	2.47±2.22	<0.001[a]
FT3 (pmol/L)	4.67±0.78	4.80±0.59	0.269[c]
FT4 (pmol/L)	15.54±3.54	15.61±3.03	0.766[a]
TG (mmol/L)	1.19±0.75	1.28±0.77	0.199[a]
CHOL (mmol/L)	4.31±1.00	4.75±0.79	<0.001[a]
HDL (mmol/L)	1.19±0.31	1.25±0.31	0.193[a]
LDL (mmol/L)	2.51±0.86	2.62±0.64	0.056[a]
FBG (mmol/L)	6.21±1.41	5.61±0.80	0.024[a]
Cortisol (nmol/L)	355.11±142.65	323.60±114.15	0.136[c]
Uric acid (μmol/L)	361.45±116.45	356.46±89.57	0.763[c]
HR (times/minute)	83.47±17.21	68.04±10.22	<0.001[a]
QRS complex (ms)	90.68±10.12	96.29±10.63	0.001[a]
PR interval (ms)	144.07±20.97	153.95±17.95	0.002[c]
QTc (ms)	361.33±28.73	392.43±25.94	<0.001[c]

SD = Standard Deviation; BMI= Body Mass Index; TSH=Thyroid Stimulating Hormone; FT3= Free Triiodothyronine; FT4=Free Thyroxine; TG=triglyceride; CHOL=Cholesterol; HDL=High D ensity Lipoprotein; LDL=Low Density Lipoprotein; FBG=Fasting Blood Glucose; HR=Heart Rat e.

[a] The *p*-values were obtained by a Mann-Whitney U test.

[b] The *p*-value for sex distribution was obtained by a Chi-Square test.

[c] The *p*-values were obtained by a Two sample T test.

Table S3. Comparison of patients and healthy controls in psychological status

Variables	Patients (Mean ± SD, n=77)	Controls (Mean ± SD, n=83)	*p*-value
**BRMS**	15.45±9.19	0.06±0.45	<0.001[a]
**HAMD**	13.42±9.20	2.80±3.88	<0.001[a]
**HAMA**	9.36±7.31	2.27±2.92	<0.001[a]
**SDSS**	4.08±3.26	0.02±0.16	<0.001[a]
**SSRS**			

Objective support score	9.49±4.14	10.94±3.15	0.016[b]
Subjective support score	19.45±5.46	23.32±5.54	<0.001[a]
Utilization of support	7.74±2.57	8.59±1.95	0.031[a]
total score	36.76±9.66	42.86±8.77	<0.001[b]
**SCSQ**			
Active coping	23.95±8.77	23.89±6.20	0.622[a]
Passive coping	9.11±5.03	7.57±4.14	0.041[a]

SD = Standard Deviation; BRMS = Bech-Rafaelsen Mania Rating Scale; HAMD = Hamilton Depression Rating Scale; HAMA =Hamilton Anxiety Rating Scale; SDSS = Social Disability Screening Schedule; SSRS = Social Support Rating Scale; SCSQ = Simplified Coping Style Questionnaire.

[a] The p-values were obtained by a Mann-Whitney U test.

[b] The *p*-values were obtained by two sample *t*-tests.

Table S4. Comparison of patients and healthy controls in cognitive status

Tests	Patients (Mean ± SD, n=77)	Controls (Mean ± SD, n=83)	*p*-value
**RBANS**			
List learning	21.95±6.14	27.70±6.42	<0.001[a]
Story memory	9.19±4.65	13.88±5.68	<0.001[b]
Figure copy	17.38±3.06	17.64±2.70	0.533[a]
Semantic fluency	16.47±4.44	18.75±4.53	0.003[a]
Digit span	12.66±2.50	13.79±2.51	0.002[a]
Coding	40.73±12.73	50.88±13.78	<0.001[b]
List recall	4.45±2.55	6.72±2.85	<0.001[a]
List recognition	18.70±1.67	19.99±3.48	<0.001[a]
Story recall	4.42±2.95	7.33±3.41	<0.001[a]
Figure recall	10.64±4.83	14.67±4.18	<0.001[a]
Total score	156.28±32.23	191.35±35.98	<0.001[a]
**SCWT**			
At	61.11±14.96	50.83±11.13	<0.001[a]
Bt	103.44±29.43	76.04±18.86	<0.001[a]
Ct	178.03±60.68	133.95±37.29	<0.001[a]
(C-B)/A	1.24±0.64	1.15±0.50	0.675[a]
C-2B+100	72.10±41.78	82.26±30.09	0.016[a]
**Error reaction**			
Ae (missay)	1.14±1.84	0.15±0.50	<0.001[a]
Ae (correction)	1.41±1.81	0.80±1.02	0.100[a]
Ae (block)	0.22±0.63	0.14±0.41	0.455[a]
Ae (total)	2.77±2.89	1.09±1.22	<0.001[a]
Be (missay)	1.78±2.95	0.67±1.27	0.011[a]
Be (correction)	2.68±2.62	1.88±1.76	0.105[a]
Be (block)	1.18±1.95	0.80±1.62	0.076[a]

Be (total)	5.68±4.98	3.34±3.34	0.001[a]
Ce (missay)	3.03±4.18	1.84±2.75	0.089[a]
Ce (correction)	4.15±4.31	3.06±2.90	0.200[a]
Ce (block)	3.66±3.99	2.09±2.99	0.004[a]
Ce (total)	10.85±8.09	6.99±6.58	<0.001[a]
(C-B)/A	1.99±2.76	1.51±2.55	0.053[a]
C-2B+100	99.50±7.14	100.44±5.16	0.337[a]
**WCST**			
Categories completed	3.70±1.94	5.20±1.40	<0.001[a]
Total number of trials administered	46.84±4.07	44.01±3.78	<0.001[a]
Correct response	26.99±9.74	33.90±6.24	<0.001[a]
Errors	19.55±9.68	10.11±8.53	<0.001[a]
Perseverative response	6.61±7.36	2.68±4.23	<0.001[a]
Perseverative errors	3.81±3.73	0.80±1.35	<0.001[a]
**EEM**			
NEF	20.51±6.59	26.53±5.21	<0.001[a]
RSS1	1.68±0.80	2.35±0.95	<0.001[a]
RSS2	1.68±0.90	2.18±0.91	<0.001[a]
RSS total	3.51±1.67	4.53±1.53	<0.001[a]
D	6.00±1.22	4.60±1.42	<0.001[b]
**ERP**			
N100	108.85±15.52	106.94±27.09	0.137[a]
P200	172.83±38.96	175.07±20.85	0.600[a]
N200	233.30±44.45	213.27±41.17	0.005[a]
P300	312.91±39.50	292.93±41.31	0.019[a]

SD = Standard Deviation; RBANS = Repeatable Battery for the Assessment of Neuropsychological Status; SCWT=Stroop color word test; WCST = Wisconsin card sorting test; EEM = Exploratory eye movement; NEF = number of eye fixation; RSS = responsive search score; D=Discriminant analysis; ERP=Event related potential.

[a] The p-values were obtained by a Mann-Whitney U test.

[b] The *p*-values were obtained by two sample *t*-tests.

Table S5. Characteristics of patients who finished the follow-up.

Variables	Pre-treatment (Mean ± SD, n=38)	Post-treatment (Mean ± SD, n=38)	*p*
Age (years)	28.82±8.12		
Gender (male/female)	11/27		
Years of education (years)	12.97±3.34		
BMI (kg/m^2)	24.86±4.73		
TSH (mIU/L)	1.90±1.44	3.83±3.41	<0.001[a]
FT3 (pmol/L)	4.70±0.71	4.14±0.86	0.001[a]
FT4 (pmol/L)	15.87±3.65	11.95±3.35	<0.001[b]
TG (mmol/L)	1.34±0.86	1.94±1.00	<0.001[a]

CHOL (mmol/L)	4.50±0.95	4.91±0.89	0.001[b]
HDL (mmol/L)	1.19±0.30	1.25±0.31	0.260[b]
LDL (mmol/L)	2.67±0.86	2.80±0.78	0.072[a]
FBG (mmol/L)	6.26±1.49	5.66±1.48	0.049[a]
Cortisol (nmol/L)	378.48±149.11	278.99±115.65	0.002[b]
Uric acid (μmol/L)	387.46±134.41	402.78±122.98	0.464[a]
HR (times/minute)	82.43±17.26	80.41±12.99	0.889[a]
QRS complex (ms)	89.95±9.83	95.32±11.02	<0.001[b]
PR interval (ms)	143.27±19.86	152.08±15.15	0.001[b]
QTc (ms)	363.46±30.11	374.03±29.42	0.074[b]

SD = Standard Deviation; BMI= Body Mass Index; TSH=Thyroid Stimulating Hormone; FT3= Free Triiodothyronine; FT4=Free Thyroxine; TG=triglyceride; CHOL=Cholesterol; HDL=High D ensity Lipoprotein; LDL=Low Density Lipoprotein; FBG=Fasting Blood Glucose; HR=Heart Rat e.

[a] The *p*-values were obtained by Wilcoxon signed-rank tests

[b] The *p*-values were obtained by paired *t*-tests.

Table S6. Characteristics of patients who finished the follow-up.

Variables	Pre-treatment (Mean ± SD, n=38)	Post-treatment (Mean ± SD, n=38)	*p*
**BRMS**	14.76±9.68	3.34±3.70	<0.001[a]
**HAMD**	14.43±8.55	6.08±5.80	<0.001[a]
**HAMA**	9.74±7.22	4.74±4.75	<0.001[a]
**SDSS**	4.11±3.52	2.18±2.73	0.005[a]
**SSRS**			
Objective support score	9.16±3.99	8.65±2.78	0.518[a]
Subjective support score	18.84±4.71	19.68±3.71	0.269[b]
Utilization of support	8.32±2.56	8.14±2.31	0.636[a]
total score	36.32±8.57	36.46±6.14	0.851[b]
**SCSQ**		±	
Active coping	24.82±7.75	21.21±7.14	0.015[b]
Passive coping	8.76±4.62	7.63±3.93	0.192[b]

SD = Standard Deviation; BRMS = Bech-Rafaelsen Mania Rating Scale; HAMD = Hamilton Depression Rating Scale; HAMA =Hamilton Anxiety Rating Scale; SDSS = Social Disability Screening Schedule; SSRS = Social Support Rating Scale; SCSQ = Simplified Coping Style Questionnaire.

[a] The *p*-values were obtained by Wilcoxon signed-rank tests

[b] The *p*-values were obtained by paired *t*-tests.

Table S7. Characteristics of patients who finished the follow-up.

Variables	Pre-treatment (Mean ± SD, n=38)	Post-treatment (Mean ± SD, n=38)	*p*
**RBANS**			
List learning	22.74±6.32	27.03±6.10	<0.001[a]
Story memory	9.95±5.08	11.05±5.01	0.097[a]
Figure copy	17.61±3.19	16.86±2.95	0.141[b]

Semantic fluency	17.58±4.37	17.46±4.25	0.850[b]
Digit span	13.13±2.24	12.73±2.56	0.180[b]
Coding	43.08±10.24	45.00±11.80	0.171[a]
List recall	4.58±2.65	6.51±2.19	<0.001[b]
List recognition	19.03±1.17	19.59±0.64	0.004[b]
Story recall	5.32±2.95	5.59±3.12	0.402[a]
Figure recall	11.11±4.73	11.51±4.82	0.503[a]
Total scores	164.11±30.43	173.35±29.75	0.001[a]
**SCWT**			
At	60.47±15.37	58.14±13.32	0.148[a]
Bt	104.18±26.55	99.12±24.16	0.125[a]
Ct	177.20±54.14	164.18±34.14	0.213[b]
(C-B)/A	1.21±0.50	1.15±0.47	0.738[a]
C-2B+100	70.65±39.27	62.42±33.34	0.678[b]
**Error reaction**			
Ae(missay)	1.13±2.00	0.41±0.87	0.075[b]
Ae(correction)	1.21±1.76	0.86±1.36	0.139[b]
Ae(block)	0.13±0.34	0.30±0.62	0.184[b]
Ae(total)	2.47±2.69	1.41±1.89	0.037[b]
Be(missay)	1.84±3.19	1.24±1.34	0.889[b]
Be(correction)	2.47±2.06	2.35±2.25	0.635[b]
Be(block)	1.24±1.81	1.78±2.29	0.365[b]
Be(total)	5.63±4.09	4.86±4.02	0.315[a]
Ce(missay)	2.92±3.89	2.24±2.59	0.693[b]
Ce(correction)	4.34±4.21	4.57±4.03	0.952[b]
Ce(block)	3.61±3.61	3.92±2.79	0.693[b]
Ce(total)	10.89±7.49	15.22±22.91	0.658[b]
(C-B)/A	2.10±2.72	3.05±3.35	0.816[b]
C-2B+100	99.68±6.54	99.80±5.78	0.918[b]
**WCST**			
Categories completed	4.00±1.82	4.25±1.66	0.487[b]
Total number of trials administered	47.39±1.60	47.56±1.30	0.553[b]
Correct response	28.26±9.60	30.39±8.03	0.168[b]
Errors	18.55±10.60	17.11±8.30	0.300[b]
Perseverative response	7.55±8.59	4.00±3.86	0.008[b]
Perseverative errors	3.71±2.24	3.14±2.30	0.203[b]
**EEM**			
NEF	21.17±5.64	20.72±7.29	0.911[a]
RSS1	1.64±0.76	1.44±1.01	0.313[b]
RSS2	1.94±1.01	1.88±0.79	0.733[b]
RSS total	3.86±2.06	3.28±1.33	0.244[b]
D	5.77±1.27	6.09±1.43	0.405[b]
**ERP**			

N100	107.60±16.20	102.87±18.02	0.160[b]
P200	173.08±32.56	175.54±24.77	0.385[b]
N200	234.62±35.09	233.97±29.40	0.896[b]
P300	316.78±36.55	314.22±27.71	0.906[b]

SD = Standard Deviation; RBANS = Repeatable Battery for the Assessment of Neuropsychological Status; SCWT=Stroop color word test; WCST = Wisconsin card sorting test; EEM = Exploratory eye movement; NEF = number of eye fixation; RSS = responsive search score; D=Discriminant analysis; ERP=Event related potential.

[a] The *p*-values were obtained by paired *t*-tests.

[b] The *p*-values were obtained by Wilcoxon signed-rank tests

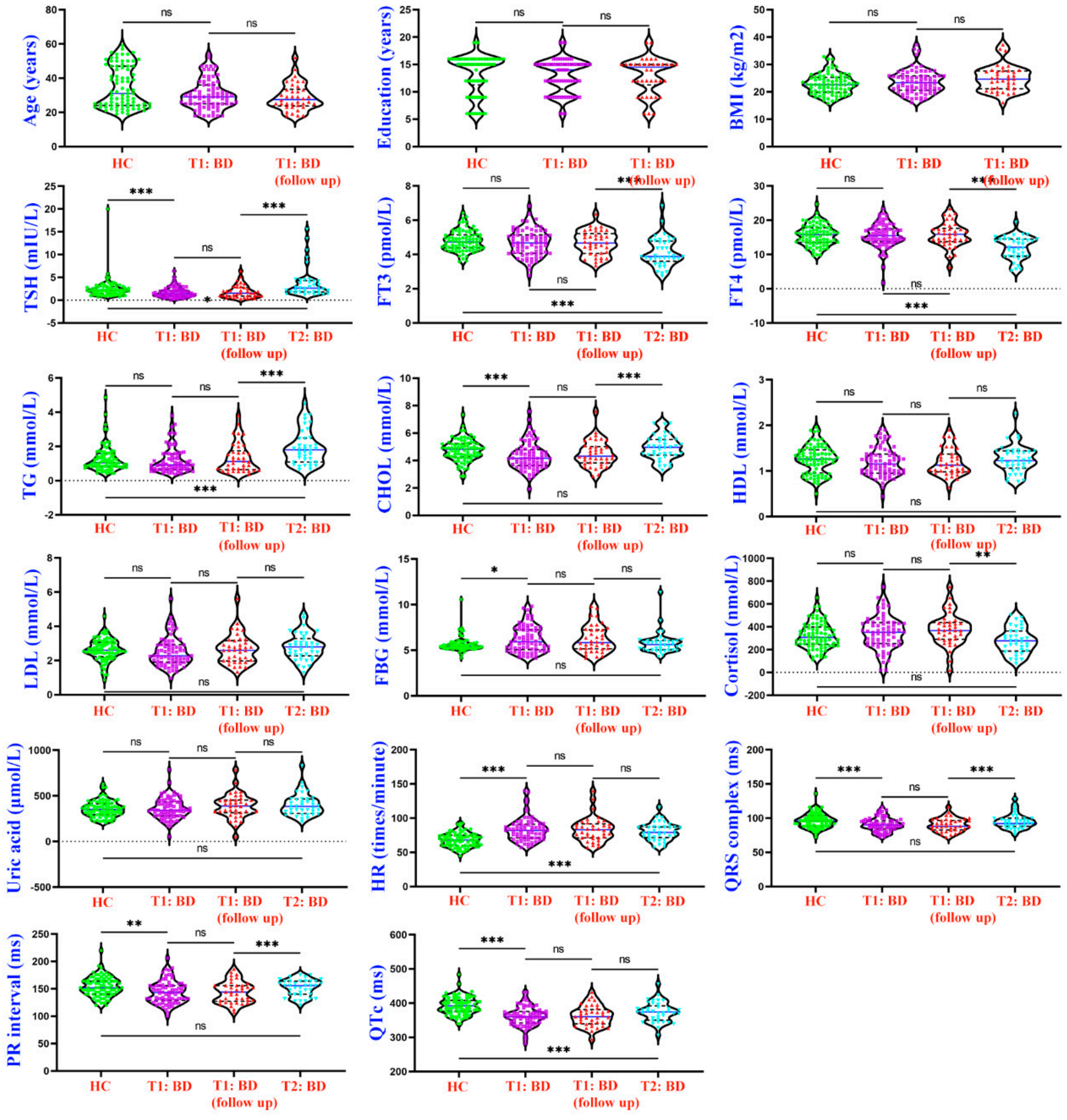

Figure S1: Between group differences in demography information and clinical variables. ns represents no difference; * represents p <0.05; ** represents p <0.01; *** represents p <0.001; **** represents p <0.0001. BMI= Body Mass Index; TSH=Thyroid Stimulating Hormone; FT3=Free Triiodothyronine; FT4=Free Thyroxine; TG=triglyceride; CHOL=Cholesterol; HDL=High Density Lipoprotein; LDL=Low Density Lipoprotein; FBG=Fasting Blood Glucose; HR=Heart Rate.

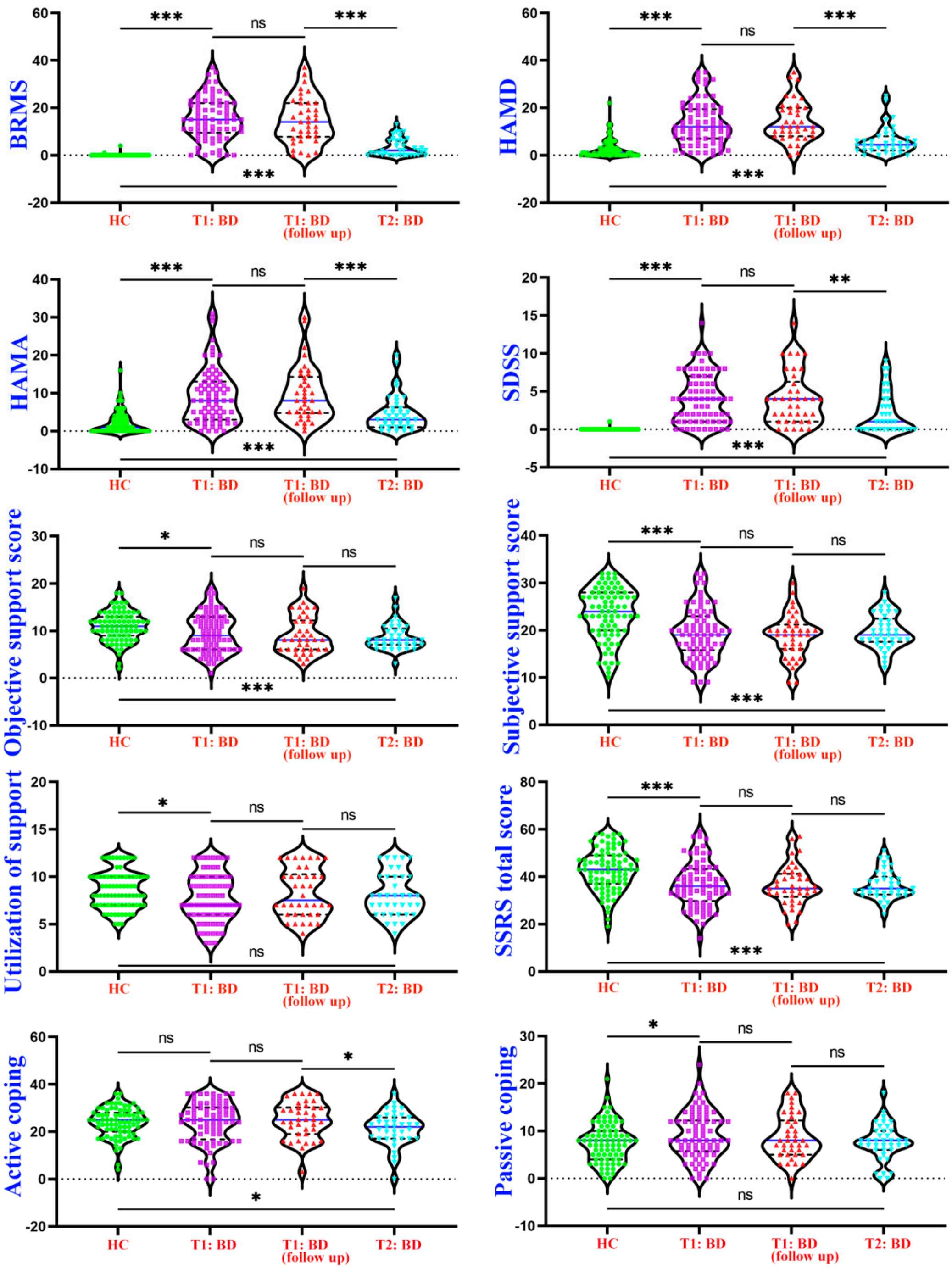

Figure S2: Between group differences in BRMS, HAMD, HAMA, SDSS, SSRS, and SCSQ. ns represents no difference; * represents $p < 0.05$; ** represents $p < 0.01$; *** represents $p < 0.001$. BRMS = Bech-Rafaelsen Mania Rating Scale; HAMD = Hamilton Depression Rating Scale; HAMA =Hamilton Anxiety Rating Scale; SDSS = Social Disability Screening Schedule; SSRS = Social Support Rating Scale; SCSQ = Simplified Coping Style Questionnaire.

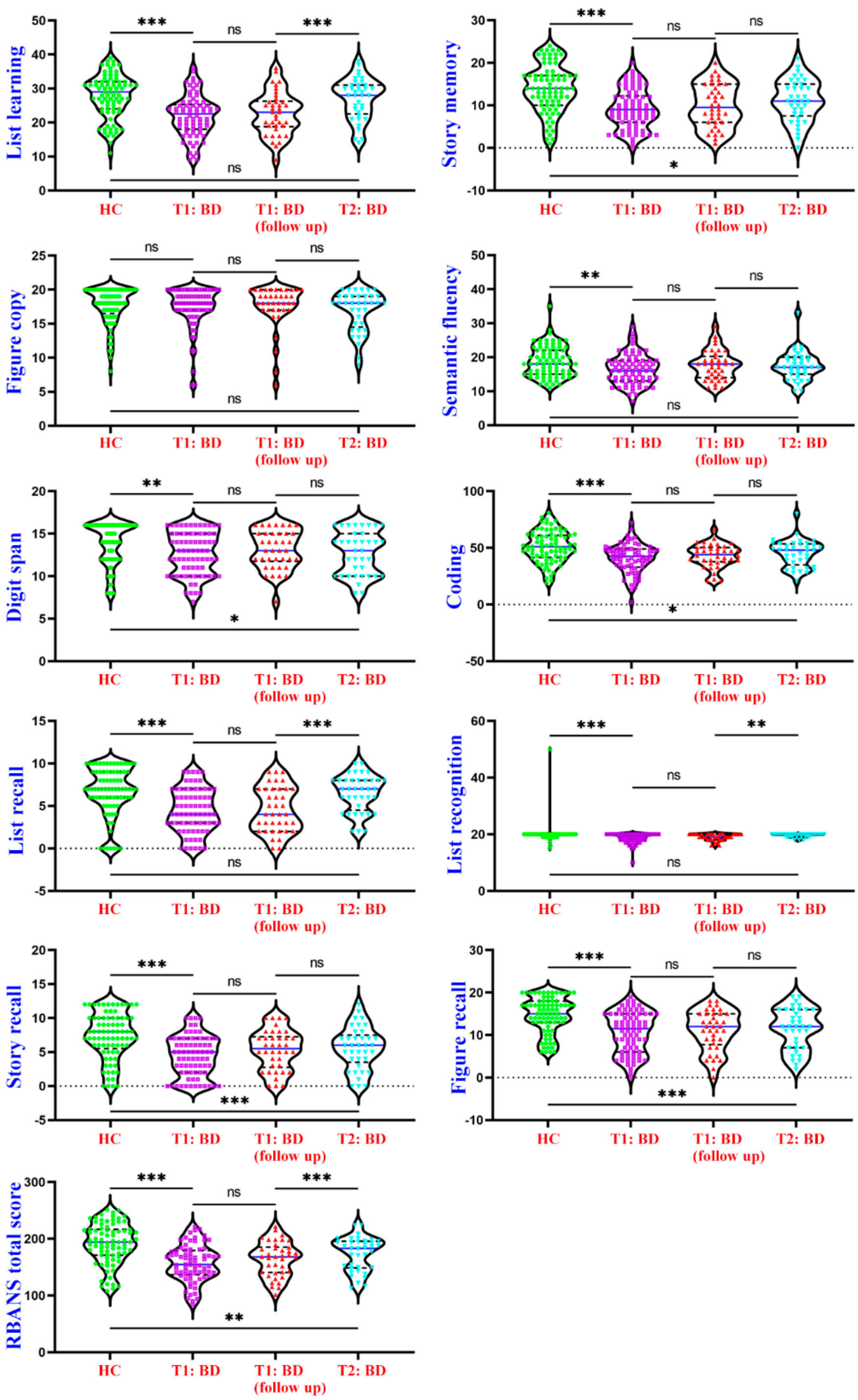

Figure S3: Between group differences in Repeatable Battery for the Assessment of Neuropsychological Status. ns represents no difference; * represents p <0.05; ** represents p <0.01; *** represents p <0.001.

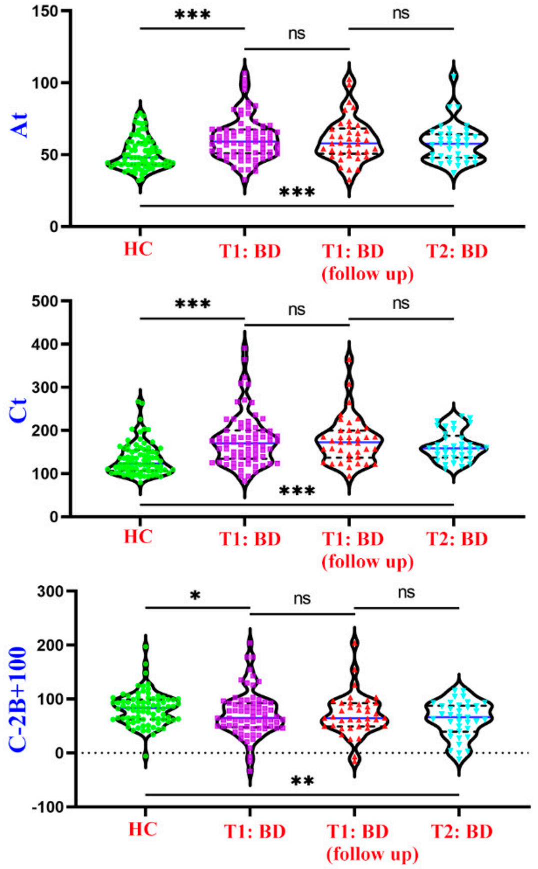

Figure S4: Between group differences in Stroop Color Word Test. ns represents no difference; * represents p <0.05; ** represents p <0.01; *** represents p <0.001.

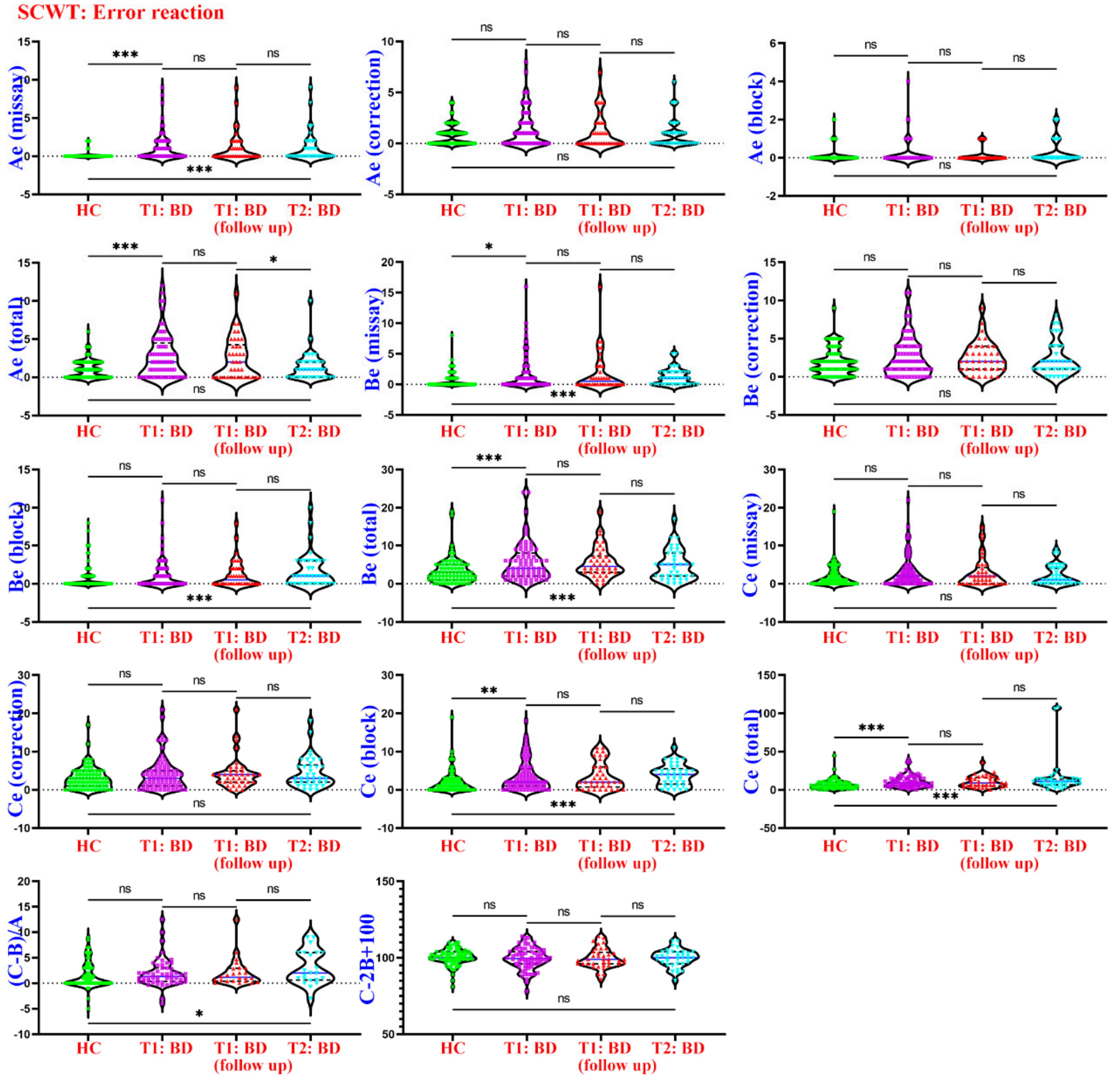

Figure S5: Between group differences in Error Reaction of Stroop Color Word Test. ns represents no difference; * represents p <0.05; ** represents p <0.01; *** represents p <0.001.

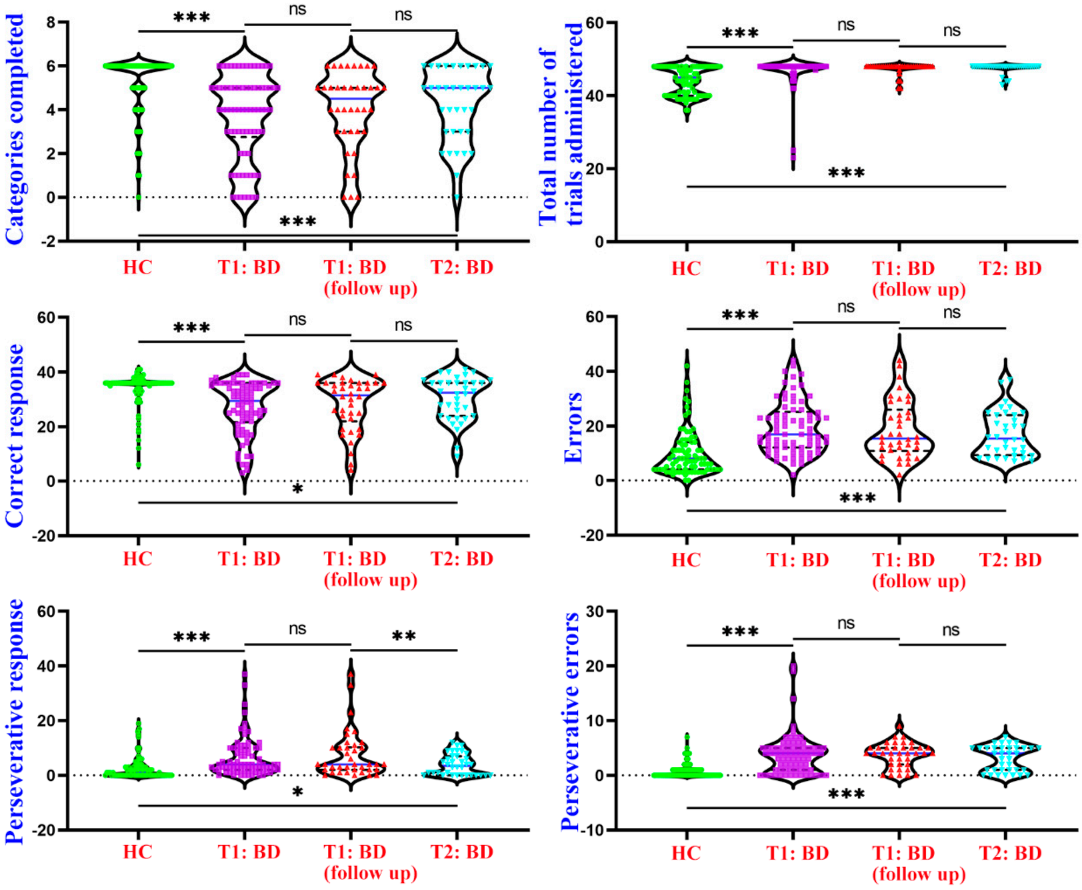

Figure S6: Between group differences in Wisconsin Card Sorting Test. ns represents no difference; * represents p <0.05; ** represents p <0.01; *** represents p <0.001.

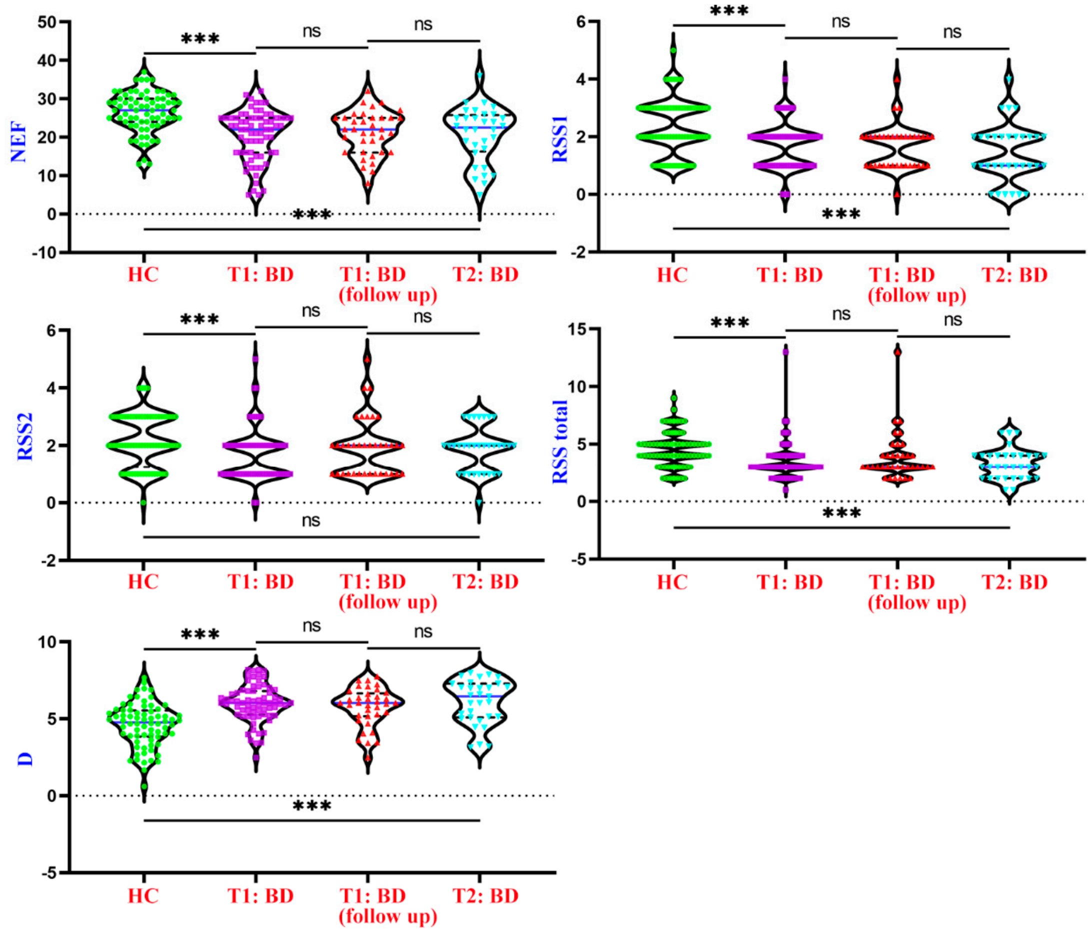

Figure S7: Between group differences in Exploratory Eye Movement. ns represents no difference; * represents p <0.05; ** represents p <0.01; *** represents p <0.001. NEF = number of eye fixation; RSS = responsive search score; D=Discriminant analysis.

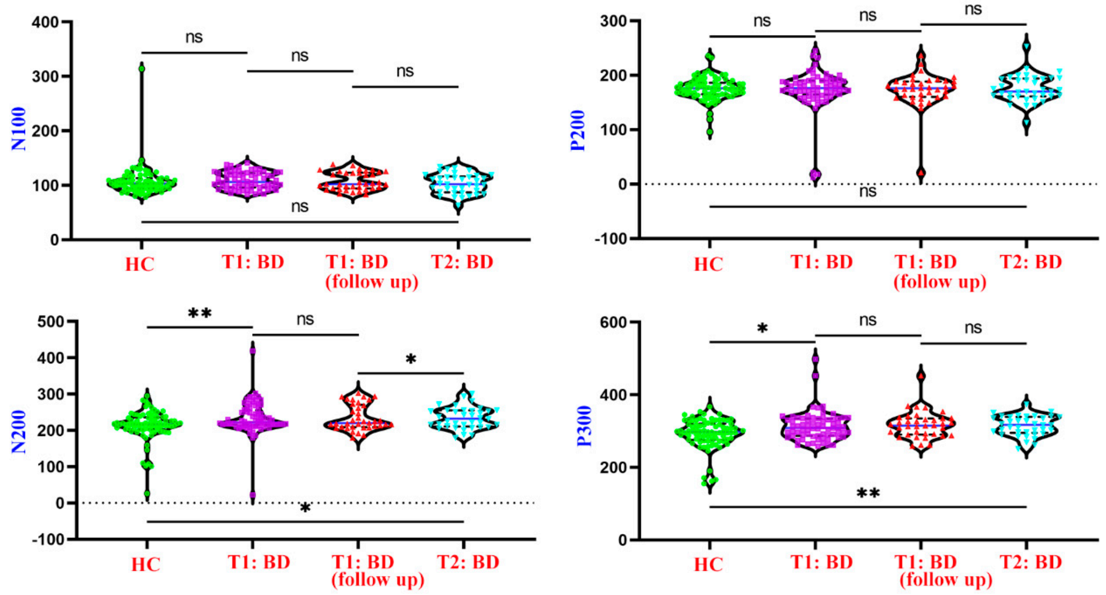

Figure S8: Between group differences in Event Related Potential. ns represents no difference; * represents p <0.05; ** represents p <0.01; *** represents p <0.001.

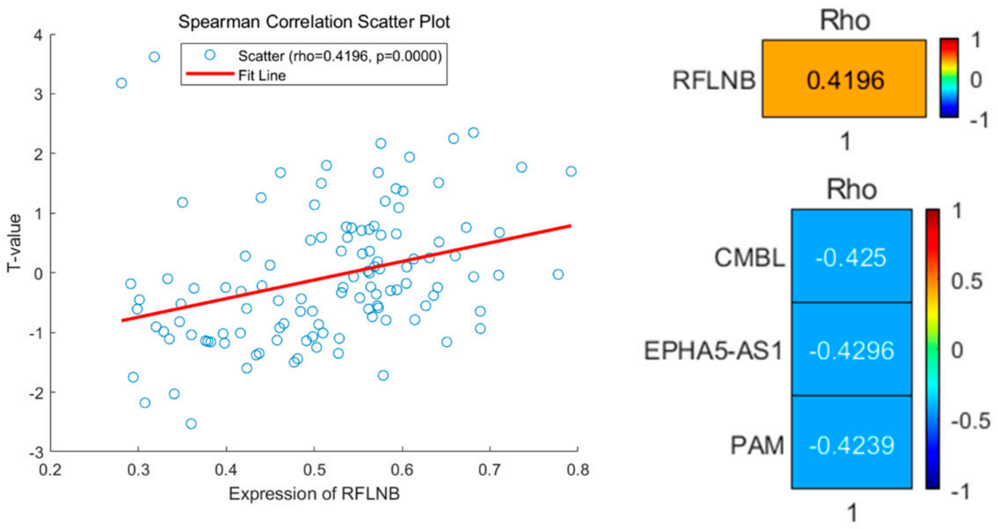

Figure S9. Spearman correlation scatter plots show the correlations with the maximal p-value in associations between gene expression profiles and functional connectivity alterations from the left lateral sensorimotor network to the rest of brain. The two correlation heatmaps separately illustrate the positive or negative correlation coefficients associated between gene expression profiles and functional connectivity alterations from the left lateral sensorimotor network to the rest of the brain.

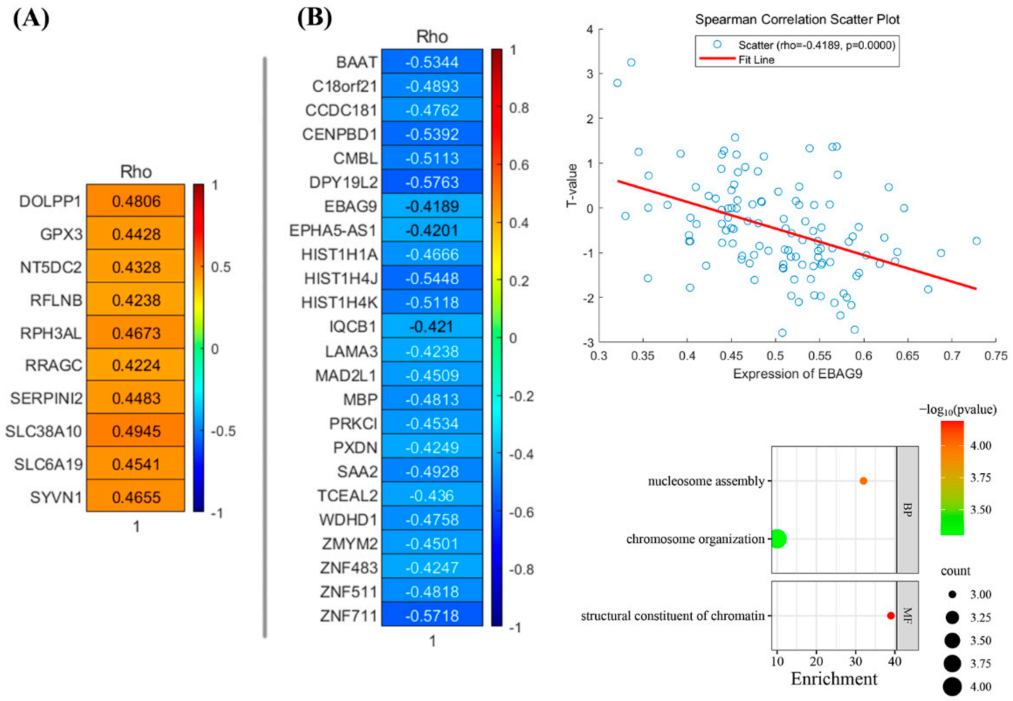

Figure S10. The two correlation heatmaps separately illustrate the positive (Part A) or negative (Part B) correlation coefficients between gene expression profiles and functional connectivity alterations from the right lateral sensorimotor network to the rest of the brain. Spearman correlation scatter plots display the correlations with the maximal p-value in associations between gene expression profiles and functional connectivity alterations from the right lateral sensorimotor network to the rest of the brain. Additionally, the bubble diagrams depict the enrichment results of genes whose expression levels were negatively correlated with functional connectivity alterations (Part B) from the right lateral sensorimotor network

to the rest of the brain regions.

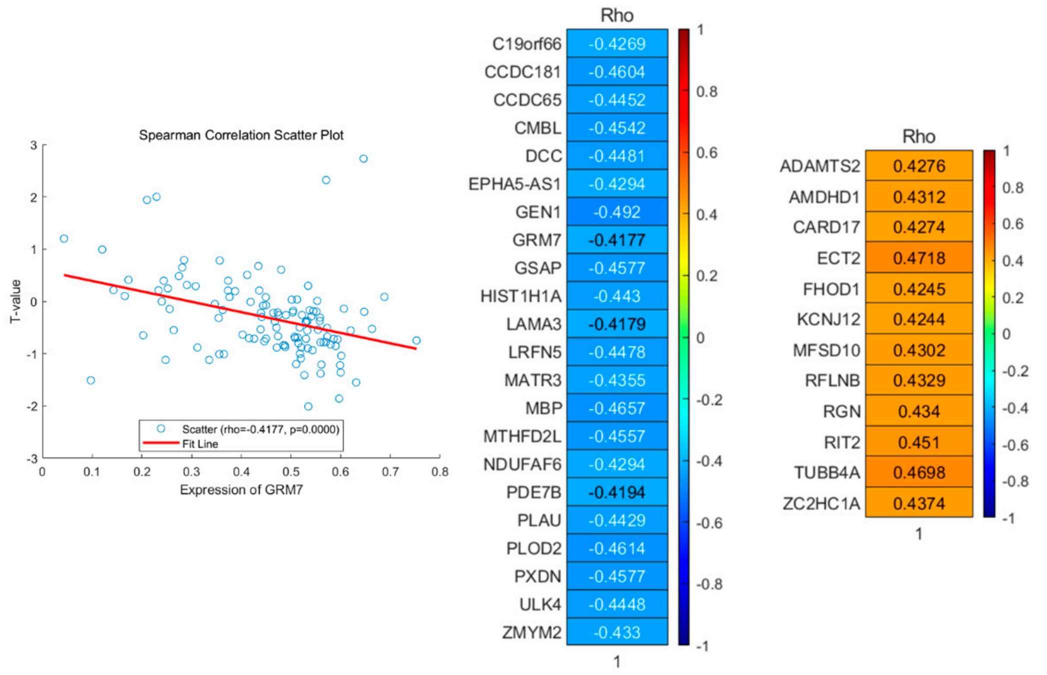

Figure S11. Spearman correlation scatter plots show the correlations with the maximal p-value in associations between gene expression profiles and functional connectivity alterations from the superior sensorimotor network to the rest of brain. The two correlation heatmaps separately illustrate the positive or negative correlation coefficients associated between gene expression profiles and functional connectivity alterations from the superior sensorimotor network to the rest of the brain.

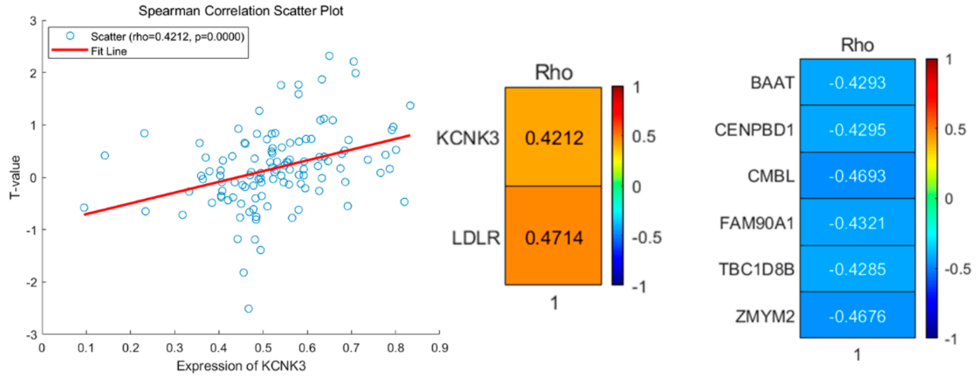

Figure S12. Spearman correlation scatter plots show the correlations with the maximal p-value in associations between gene expression profiles and functional connectivity alterations from the occipital visual network to the rest of brain. The two correlation heatmaps separately illustrate the positive or negative correlation coefficients associated between gene expression profiles and functional connectivity alterations from the occipital visual network to the rest of the brain.

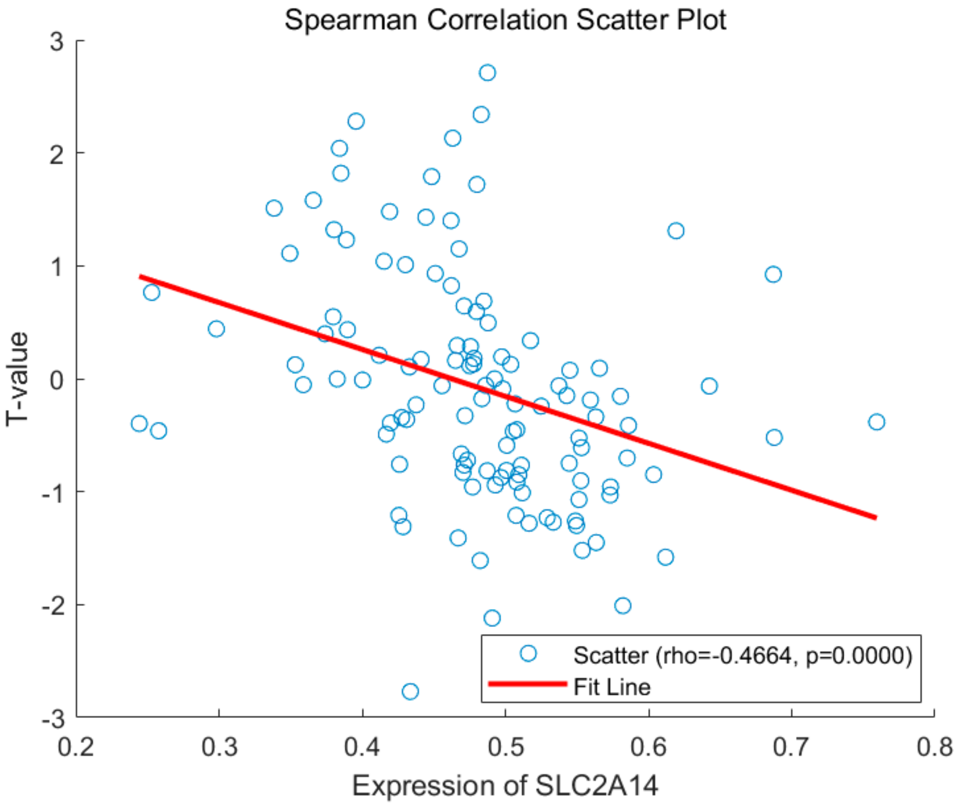

Figure S13. The Spearman correlation scatter plot shows the correlations between the expression level of SLC2A14 and functional connectivity alterations from the left lateral visual network to the rest of the brain.

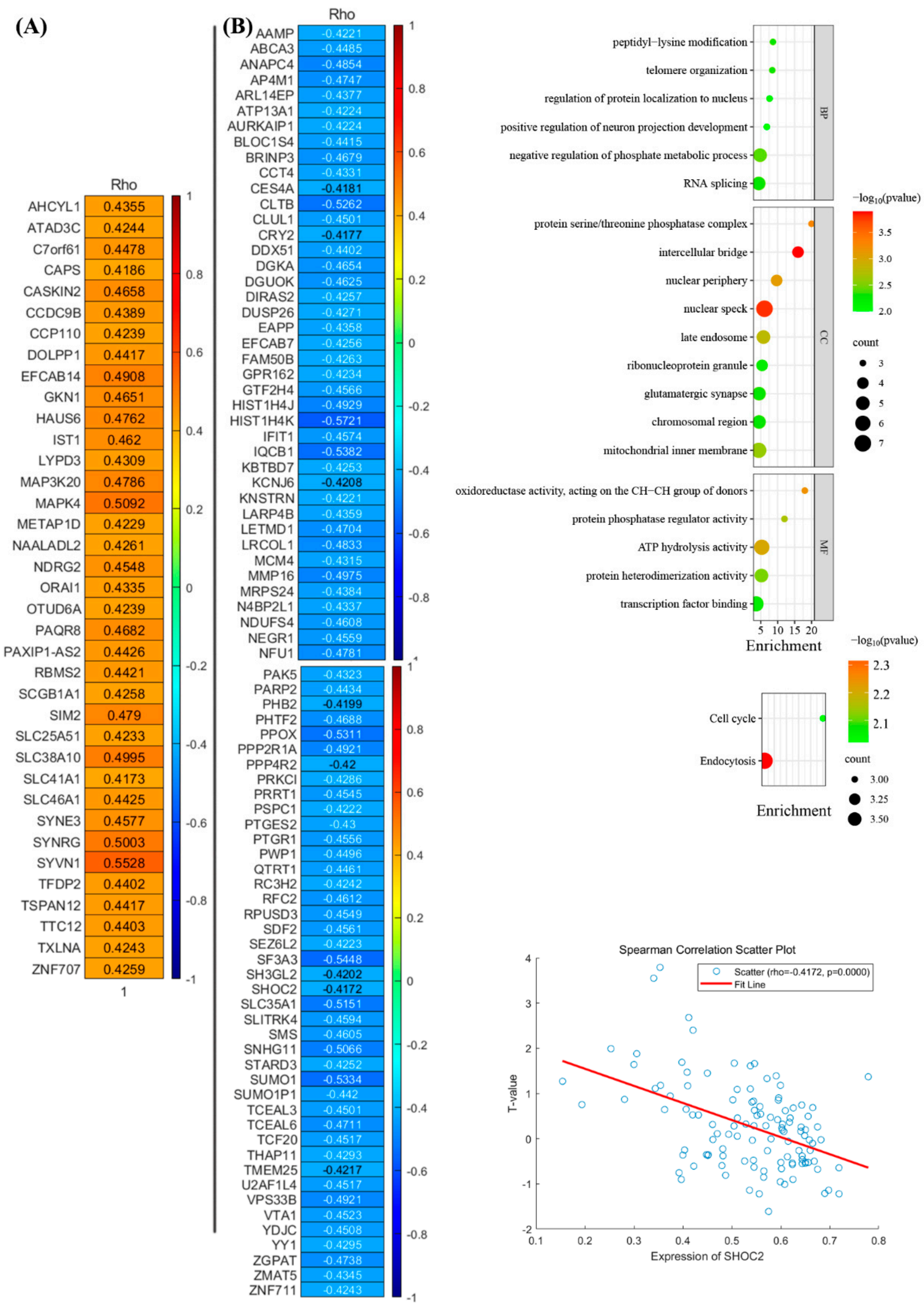

Figure S14. The two correlation heatmaps separately illustrate the positive (Part A) or negative (Part B) correlation coefficients between gene expression profiles and functional connectivity alterations from the right lateral visual network to the rest of the brain. Spearman correlation scatter plots display the correlations with the maximal p-value in associations between gene expression profiles and functional connectivity alterations from the right lateral visual network to the rest of the brain. Additionally, the bubble diagrams depict the enrichment results of genes whose expression levels were negatively correlated with functional connectivity alterations (Part B) from the right lateral visual network to the

rest of the brain regions.
